# A diagnostic performance study of multiplex polymerase chain reaction-based targeted next−generation sequencing for the accurate identification of *Mycobacterium* tuberculosis in bronchoalveolar lavage fluid

**DOI:** 10.3389/fcimb.2026.1807848

**Published:** 2026-06-26

**Authors:** Hansheng Wang, Dan Cheng, Xin Qian, Xianru Xia, Biyu Chen, Chenglin Wu, Fang Wang, Sijia Guo, Yujie Gao, Yi Wu, Tao Ren, Yijun Tang, Meifang Wang

**Affiliations:** 1 Department of Pulmonary and Critical Care Medicine, Shiyan Key Laboratory of Major Chronic Respiratory Disease, Taihe Hospital, Hubei University of Medicine, Shiyan, Hubei, China; 2 Department of Blood Transfusion Medicine, Taihe Hospital, Hubei University of Medicine, Shiyan, Hubei, China; 3 Clinical Molecular Diagnosis Center, Taihe Hospital, Hubei University of Medicine, Shiyan, Hubei, China

**Keywords:** bronchoalveolar lavage fluid (BALF), drug resistance detection, mycobacterium tuberculosis, targeted next-generation sequencing (TNGS), Xpert MTB/RIF

## Abstract

**Objective:**

To evaluate the diagnostic performance of multiplex PCR-based targeted next-generation sequencing (mp-tNGS) for detecting *Mycobacterium tuberculosis* (MTB) in bronchoalveolar lavage fluid (BALF) from patients with suspected pulmonary tuberculosis (PTB).

**Methods:**

This prospective study enrolled 188 patients. BALF samples were tested in parallel by AFB smear, culture, Xpert MTB/RIF, and mp-tNGS. Diagnostic performance was assessed against a composite clinical reference standard; phenotypic drug susceptibility testing (pDST) was additionally compared in a subset of 35 patients.

**Results:**

Among the cohort, 116 patients were diagnosed with PTB. The sensitivities of AFB smear, culture, Xpert MTB/RIF, and mp-tNGS were 19.8%, 33.6%, 73.3%, and 83.6%, respectively; specificities were 91.7%, 100%, 98.6%, and 97.2%. mp-tNGS demonstrated the highest diagnostic accuracy (88.8%) and the strongest agreement with the reference standard (κ = 0.868, *p* < 0.001); Subgroup analysis using microbiological reference standard confirmed comparable performance (sensitivity 83.8%, specificity 97.2%). The area under the ROC curve for mp-tNGS was 0.88, significantly outperforming AFB smear (0.56), culture (0.67), and Xpert MTB/RIF (0.81). In the resistance subset, mp-tNGS showed 100% sensitivity and specificity for rifampicin resistance (all *rpoB* S450L mutations) and 75% sensitivity with 100% specificity for isoniazid resistance (*katG* S315T mutations), identifying five probable multidrug-resistant cases. Xpert MTB/RIF detected rifampicin resistance with 75.0% sensitivity and 96.3% specificity.

**Conclusion:**

The mp-tNGS assay provides a superior combination of rapid turnaround time (~12 hours) and high diagnostic accuracy for MTB detection in BALF. Beyond pathogen identification, it offers rapid and preliminary genotypic resistance screening, supporting early suspicion of multidrug resistance, while phenotypic confirmation remains essential. This makes mp-tNGS a highly effective tool for the rapid, accurate diagnosis and preliminary resistance screening of PTB.

## 1 Introduction

Tuberculosis (TB) is an airborne infectious disease caused by the bacillus *Mycobacterium tuberculosis* (MTB) complex, primarily affecting the lungs (pulmonary TB) and characterized by chronic systemic wasting (World health organization global tuberculosis report, 2024; Global, regional, and national age-specific progress towards the 2020 milestones of the WHO End TB Strategy: a systematic analysis for the Global Burden of Disease Study 2021, 2024; Khaing et al., 2024). It is estimated that approximately one-quarter of the global population has been infected with the TB pathogen (Houben and Dodd, 2016). According to the 2024 World Health Organization (WHO) Global Tuberculosis Report (World health organization global tuberculosis report, 2024), in 2023, 10.8 million people developed the disease, corresponding to an incidence rate of 134 per 100,000 population. The distribution of cases was 55% among males, 33% among females, and 12% among children and adolescents. For three consecutive years prior to 2023, COVID-19 had displaced TB as the leading infectious cause of death worldwide. However, TB is estimated to have regained this position in 2023, with a mortality burden nearly double that of HIV/AIDS. TB remains a persistent global health challenge, with an annual case count exceeding 10 million—a figure that has been rising since 2021 (World health organization global tuberculosis report, 2024). Accurate diagnosis and differential diagnosis of tuberculosis are essential for effective disease control and prevention, particularly in non-tuberculosis specialized settings. Early detection not only enables timely and appropriate treatment, thereby improving patient prognosis, but also helps interrupt transmission chains and reduce overall disease incidence.

Conventional diagnostic methods for pulmonary TB (PTB) each present significant limitations. Acid-fast bacilli (AFB) smear microscopy, while rapid and inexpensive, suffers from low sensitivity (30-60%) and cannot distinguish MTB complex from nontuberculous mycobacteria (NTM), leading to potential misdiagnosis and inappropriate treatment (World health organization global tuberculosis report, 2024; Khaing et al., 2024). Mycobacterial culture, the traditional gold standard for species identification and drug susceptibility testing (DST), requires weeks to yield results due to the slow growth of MTB, delaying crucial therapeutic decisions (Houben and Dodd, 2016). Although the tuberculin skin test (TST) has been used in tuberculosis diagnosis for over a century, it cannot reliably differentiate between latent TB infection (LTBI), active TB disease, and prior Bacillus Calmette–Guérin vaccination (Gong and Wu, 2021). Similarly, immunological techniques such as the interferon-gamma release assays (IGRAs) are valuable auxiliary diagnostic tools, their clinical utility in disease management is limited by a critical inability to distinguish between active tuberculosis and LTBI (Gong and Wu, 2021). The Xpert MTB/RIF assay, a nucleic acid amplification-based rapid test endorsed by the WHO in 2011, has become a widely adopted tool for diagnosing tuberculosis and detecting rifampicin resistance (Policy Statement: Automated Real-Time Nucleic Acid Amplification Technology for Rapid and Simultaneous Detection of Tuberculosis and Rifampicin Resistance: Xpert Mtb/Rif System, 2011). Its advantages—ease of use, full automation, and minimal need for dedicated PCR facilities—have promoted widespread adoption, especially in resource-limited settings. However, its clinical utility is limited by low sensitivity in smear-negative and paucibacillary specimens (e.g., ~73% in BALF) (Maynard-Smith et al., 2014; Chakravorty et al., 2017; Tang et al., 2023) and by targeting only restricted genomic loci, which hinders comprehensive resistance profiling (Günther et al., 2022).

Pulmonary tuberculosis (PTB) constitutes the majority of tuberculosis cases, with a substantial and growing proportion presenting as paucibacillary disease, posing a significant diagnostic challenge that requires differentiation from other pulmonary infections (Wang et al., 2014; Tang et al., 2021). Current diagnostics, often limited to single-pathogen detection, are inadequate for this complex etiology. Metagenomic next-generation sequencing (mNGS) has emerged as a powerful, broad-spectrum tool for pathogen detection, capable of precisely identifying bacteria, fungi, viruses, and parasites—a significant advance over conventional methods (Li et al., 2023). However, its high cost and technical complexity hinder routine clinical application and widespread adoption. Recently, targeted next-generation sequencing (tNGS) has emerged as a rapid and comprehensive diagnostic tool for infectious diseases (Cabibbe et al., 2020). This technique detects hundreds of pathogens and associated drug-resistance markers in respiratory infections. tNGS typically employs one of two enrichment strategies: a super-multiplex PCR system or a probe-capture hybridization method, followed by high-throughput sequencing for precise pathogen identification (Chen Q. et al., 2024). The PCR-based approach is cost-effective but covers fewer targets, whereas the probe-capture method offers broader pathogen coverage at a higher cost (Expert consensus on the application and practice of targeted next-generation sequencing in infectious diseases, 2024; Yin et al., 2024).

Compared to mNGS, tNGS—particularly in its multiplex PCR-based form (mp-tNGS)—presents distinct advantages, including lower cost, faster turnaround time, minimal host DNA interference, and integrated detection of DNA and RNA pathogens (Miller et al., 2019). With a panel design covering over 95% of common infectious agents, mp-tNGS enables precise etiological diagnosis in complex clinical settings such as lower respiratory tract infections (Wang et al., 2025b). Recent studies further highlight its superior sensitivity over conventional methods (e.g., culture and serology) for detecting pathogens like *Aspergillus* and Pneumocystis *jirovecii* in BALF (Wang et al., 2025b; Wang et al., 2025a). Although experts recommend tNGS for patients with paucibacillary mycobacterial disease, particularly when conventional methods yield negative results on sputum samples (Expert consensus on the application and practice of targeted next-generation sequencing in infectious diseases, 2024), the diagnostic performance of mp-tNGS—specifically optimized for accurate MTB identification in BALF—still requires further rigorous clinical validation, especially in smear-negative or paucibacillary patients with suspected PTB.

Therefore, this study aimed to conduct a diagnostic performance evaluation of an mp-tNGS assay for the detection of *Mycobacterium tuberculosis* in BALF specimens. We compared its sensitivity, specificity, and concordance with clinical diagnosis against established methods, including AFB smear, Xpert MTB/RIF, and a composite clinical reference standard, in a cohort of patients suspected of having PTB.

## 2 Patients and methods

### 2.1 Participants and study design

Between January 2022 and January 2025, patients suspected of having pulmonary tuberculosis (PTB) based on comprehensive clinical assessment—including clinical presentation, radiological findings, and laboratory tests—and who underwent BALF mp-tNGS testing were enrolled. Exclusion criteria were as follows: (1) receipt of anti-tuberculosis treatment prior to testing; (2) presence of active extrapulmonary TB infection; (3) absence of a complete medical record; (4) patients had no definite diagnosis. Complete medical records were collected for all enrolled patients, including demographic characteristics, clinical parameters, radiological findings, and laboratory results, as follows: (1) underlying diseases; (2) radiographic findings; (3) laboratory results; and (4) clinical outcomes. The final diagnosis of PTB was established by two independent senior clinicians through a comprehensive assessment. This assessment integrated the patients’ clinical manifestations, laboratory and radiological findings, and their response to anti-tuberculosis treatment. Or the final diagnosis was substantiated by pathological examination and real-time quantitative PCR analysis of tissue samples, which were obtained via percutaneous lung biopsy (PTLB), medical thoracoscopic pleural biopsy (MTPB), or transbronchial biopsy (TBB). This final diagnosis served as the reference standard against which the AFB smear, culture, Xpert MTB/RIF and mp-tNGS results were compared. The study was approved by the Ethics Committee of Taihe Hospital, and written informed consent was obtained from each patient.

### 2.2 Diagnostic criteria

The final clinical diagnosis for all enrolled patients was determined by a comprehensive assessment and served as the composite clinical reference standard (CRS) for primary analysis in this study. In diagnostic accuracy studies for PTB, there is no perfect gold standard, as culture—the traditional reference—has limited sensitivity, particularly in paucibacillary specimens, and cannot serve as an absolute benchmark for detecting all true cases (Keter et al., 2023). The CRS integrates multiple diagnostic modalities and is widely accepted in tuberculosis diagnostic research. However, CRS-based designs carry a potential risk of incorporation and verification bias when the reference standard includes components that overlap with the index test or when verification testing is not applied uniformly (Keter et al., 2024). To address this concern, we additionally performed a secondary analysis using a strict microbiological reference standard (MRS) defined as positivity by mycobacterial culture or histopathology with positive MTB PCR, restricted to patients with definitive microbiological or histopathological confirmation (n = 111). The diagnostic criteria for PTB were defined as follows: a patient was diagnosed with PTB if he/she met one of the following criteria: (1) Positive culture for *Mycobacterium tuberculosis* (MTB) from BALF or sputum. (2) Histopathological examination of tissue specimens with demonstrated granulomatous inflammation and a positive PCR result for *Mycobacterium tuberculosis* complex. (3) Clinical and radiological findings highly suggestive of PTB (*e.g.*, persistent cough, weight loss, night sweats, and cavitary or nodular opacities predominantly in the upper lobes on chest CT), along with a documented clinical decision to initiate a full course of anti-tuberculosis treatment and subsequent favorable response. Patients who met criteria (1) or (2) were considered microbiologically or histopathologically confirmed PTB and were included in the MRS subgroup. Patients who met only criterion (3) were classified as clinically diagnosed PTB and were included only in the primary CRS analysis (excluded from the MRS subgroup). Non−PTB patients were those in whom PTB was ruled out by the absence of any of the above criteria and who had an alternative definitive diagnosis established by microbiological, histopathological, or clinical follow−up evaluation.

### 2.3 AFB smear and MTB culture assays

The detailed procedure of acid-fast staining is strictly according to “Manual of Clinical Microbiology” (Versalovic, 2011). Following standardized laboratory protocols, collected BALF samples were first centrifuged to pellet cells and remove debris. The supernatant was used to prepare thin smears on sterile glass slides, which were air-dried at room temperature. Ziehl–Neelsen staining was performed by gently heat-fixing the smears, applying carbol fuchsin for approximately 5 min, rinsing with water, and counterstaining with methylene blue. Meanwhile, the centrifuged sediment was resuspended and inoculated into the MGIT™ liquid culture system (Becton Dickinson, USA). Cultures were incubated for up to 8 weeks, strictly following the MGIT™ procedure manual (Siddiqi and Rueschgerdes, 2006).

### 2.4 Xpert MTB/RIF assay

GeneXpert Mycobacterium tuberculosis (MTB)/rifampicin (RIF) analysis was employed for the detection of Mycobacterium tuberculosis. The procedure, as outlined in previous studies (Kohli et al., 2018; Liu et al., 2021), was conducted using the GeneXpert Dx instrument system (model GX-XVI R2; Cepheid). This system automates sample purification, nucleic acid amplification, and sequencing of MTB/rifampicin nucleic acids. The process began with degradation, decontamination, and concentration of the sample. Then, 0.5 ml of the resuspended sediment was placed into a conical screw-cap tube, mixed with 1.5 ml of Xpert MTB/RIF sample reagent using a sterile pipette, and shaken 10–20 times. The mixture was incubated at 20–30 °C for 15 minutes, with the incubation period initiated between 5 and 10 minutes after preparation. Subsequently, the treated sample was transferred into the sample chamber of an Xpert MTB/RIF cartridge (LOT: 92008; Cepheid, USA) via a sterile pipette and loaded into the GeneXpert Dx instrument. Upon entering sample information, the system automatically carried out sample filtration and washing, released DNA through ultrasonic lysis, combined it with PCR reagents, and performed semi-nested real-time amplification to detect fluorescence signals. Test results were automatically generated by the instrument after 2 hours.

### 2.5 mp-tNGS procedures and bioinformatics analysis

#### 2.5.1 mp-tNGS workflow construction

The mp-tNGS workflow, including database integration, was established based on our previous publication (Cabibbe et al., 2020) and the study of YIN Y et al (Chen Q. et al., 2024). The panel covers 198 pathogen targets frequently encountered in clinical practice; a complete list is provided in **Supplementary Table 1**. The commercially sourced RP100™ kit contains over 300 pre-designed and optimized multiplex amplification primer sets, such as the Mycobacterium tuberculosis complex (MTBC). A PCR process was developed and optimized to ensure high-sensitivity amplification of target sequences.

#### 2.5.2 mp-tNGS nucleic acid extraction

BALF samples were mixed with an equal volume of 0.1 M DTT liquefaction reagent, followed by vortexing, shaking, and incubation for 3–5 minutes until complete liquefaction. A total of 1.3 mL of the liquefied sample was aliquoted, spiked with 13 μL of an exogenous endogenous reagent, and centrifuged at 12,000 rpm for 5 minutes. The supernatant was discarded, and 500 μL of the sample was transferred to a bead mill tube from the extraction kit. Then, 50 μL of SDS was added, and the mixture was subjected to bead-beating using a sonicator (4,700 rpm, 45 s oscillation, 20 s pause, 2 intervals; total oscillation time 135 s) for cell disruption. After disruption, the sample was centrifuged again at 12,000 rpm for 5 minutes. Nucleic acids were extracted from 400 μL (manual) or 250 μL (automated) of the supernatant using the MetaPure DNA & RNA Extraction Kit (KingCreate, Guangzhou, China), following the manufacturer’s instructions.

#### 2.5.3 mp-tNGS library construction

mp‑tNGS library construction was performed according to Zhang et al. (2024). Library preparation was performed using the RP100™ Respiratory Pathogen Microorganisms Multiplex Testing Kit (KingCreate Biotechnology Co., Ltd., Guangzhou, China). First, cDNA was synthesized from extracted nucleic acids by reverse transcription. Subsequent steps included target region enrichment, two rounds of purification, and adapter ligation to complete library construction. Nuclease-free water (Invitrogen, Waltham, MA, USA) was used as a non-template control (NTC) to monitor contamination. Library quantification was performed using the Equalbit DNA HS Assay Kit (Vazyme Biotech, Nanjing, China) on a Qubit™ 3.0/4.0 Fluorometer (Thermo Fisher Scientific, Waltham, MA, USA). All samples met the quality threshold of ≥ 0.5 ng/μL; otherwise, libraries were re-prepared. Qualified libraries were pooled in equimolar ratios, and fragment size distribution (approximately 250–350 bp) was verified using a Qsep100 fully automated nucleic acid analyzer with a Standard Cartridge Kit (S2). Finally, the pooled library was diluted, denatured, and 500 μL was loaded into a KM MiniSeqDx-CN Sequencing Kit for sequencing on the KM MiniSeq Dx-CN Platform (KingCreate, Guangzhou, China).

#### 2.5.4 Bioinformatics analysis

Sequencing data were processed using a customized bioinformatics pipeline. Raw reads underwent quality control and adapter trimming via fastp v0.20.1 (Chen et al., 2018) with default parameters. Filtered reads were aligned against a curated mp-tNGS pathogen database using Bowtie2 v2.4.1 (Langmead and Salzberg, 2012) in very-sensitive mode. The numbers of reads per 100,000 sequencing reads (RPhK) were calculated at the species and genus levels. Resistance mutations were called with a minimum depth of ≥20×, allelic frequency ≥15%, and bidirectional read support (Ou et al., 2025). For MTB identification, positivity required ≥5 specific reads (≥97% identity) and RPhK ≥1.5; reads aligning equally to MTB and NTM were assigned to NTM to prioritize specificity. Specimens with 1–4 reads were classified as “indeterminate”. In silico cross−reactivity analysis against 20 NTM genomes revealed limited homology between three IS6110 primer sets and *M. avium*/*M. intracellulare* (consistent with prior reports (Mulcahy et al., 1996; Dziadek et al., 2001a)); no other significant cross−reactivity was identified.

#### 2.5.5 Laboratory quality control for contamination prevention

To minimize contamination, the following measures were implemented. First, each run included a non-template control (NTC); runs with >2 NTC reads for any target were invalidated and repeated. Second, MTB positivity required ≥5 specific reads (≥97% identity) and RPhK ≥1.5; specimens with 1–4 reads were classified as “indeterminate” and reflexed to confirmatory testing. Third, pre- and post-PCR areas were strictly segregated, and negative controls were included per batch. Fourth, bioinformatics excluded reads with mapping quality <30 or mismatch >3%; reads aligning equally to MTB and NTM were assigned to NTM to prioritize specificity. Additionally, our laboratory participates in NGS external quality assessment (EQA) and duplicates low-level positive signals when contamination is suspected.

### 2.6 Statistical analysis

Statistical analysis was performed using SPSS software version 26.0. The normality of continuous variables was assessed with the Shapiro−Wilk test. Normally distributed data are presented as mean ± standard deviation and were compared using the independent−samples *t*−test; non−normally distributed data are expressed as median and interquartile range (25th–75th percentiles) and were compared with the Mann–Whitney U test. Categorical variables are presented as number (percentage) and were analyzed using the chi−square test. Receiver operating characteristic (ROC) curves were generated to evaluate the sensitivity, specificity, positive predictive value (PPV), and negative predictive value (NPV) of each diagnostic assay. For paired comparisons of sensitivity and specificity between two diagnostic tests (e.g., mp-tNGS *vs*. Xpert MTB/RIF), McNemar’s test for paired proportions was used, given that all four tests were performed on the same BALF sample from each patient. For each comparison, the difference in proportions (sensitivity difference or specificity difference) was calculated with its 95% confidence interval using the Wilson score method with continuity correction for matched-pair designs. ROC curves were compared using DeLong’s test for paired ROC curves (DeLong et al., 1988; Robin et al., 2011), which accounts for the correlation between AUCs derived from the same subjects. A two−tailed *p*−value <0.05 was considered statistically significant. Figures were prepared using GraphPad Prism 9 software (GraphPad, Inc., La Jolla, CA, USA).

## 3 Results

### 3.1 Demographic and clinical characteristics

**Figure 1:**
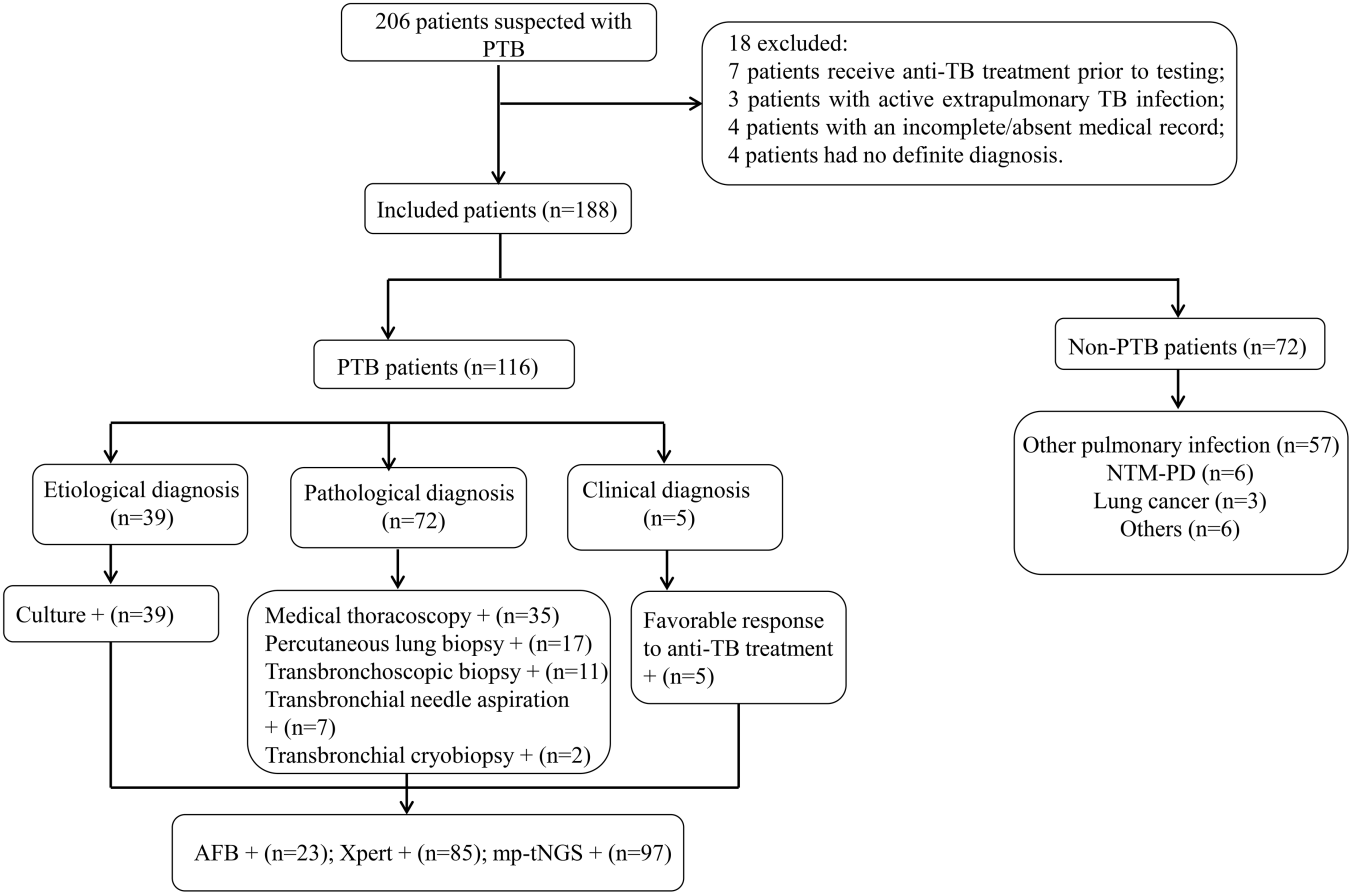
Flowchart of study population enrollment and diagnosis based on the composite clinical reference standard.

A total of 206 patients with suspected PTB were prospectively included. Based on pre-defined exclusion criteria, 18 patients were excluded, resulting in 188 patients with the CRS-based diagnosis ultimately included in the final analysis (see the study flowchart in **Figure 1**). During this prospective study period, a total of 188 consecutive patients who underwent bronchoalveolar lavage for BALF sampling and subsequent mp-tNGS testing were enrolled. Based on a composite clinical diagnostic standard, all patients were classified into either the pulmonary tuberculosis group (PTB, n=116) or the non-pulmonary tuberculosis group (Non-PTB, n=72). As detailed in **Table 1**, significant demographic differences were observed. Patients in the PTB group were significantly younger (mean age 61.2 ± 13.4 years *vs*. 65.3 ± 10.7 years, *p* = 0.000) and had a higher proportion of males (75.9% *vs*. 61.1%, *p* = 0.032). No significant differences were noted between the two groups regarding smoking or drinking history (*p >*0.05). Clinically, hemoptysis (19.0% *vs*. 48.6%, *p* = 0.000), chest tightness (25.0% *vs*. 40.3%, *p* = 0.027), and chest pain (13.8% *vs*. 27.8%, *p* = 0.018) were more common in the Non-PTB group, while cough (95.8% *vs*. 84.5%, *p* = 0.02) and expectoration (81.9% *vs*. 59.5%, *p* = 0.001) were more frequent in the Non-PTB group. Regarding underlying diseases, chronic pulmonary diseases were markedly more prevalent in the Non-PTB group (66.7% *vs*. 2.6%, *p* = 0.000), whereas solid organ malignancy was less common in the PTB group (3.4% *vs*. 11.1%, *p* = 0.037). Laboratory tests revealed that the PTB group had significantly lower levels of albumin (median 38.06 *vs*. 38.79 g/L, *p* = 0.000) and lactate dehydrogenase (median 198.7 *vs*. 179.0 U/L, *p* = 0.000), but higher levels of interleukin-6 (median 62.53 *vs*. 69.22 pg/ml, *p* = 0.000) and hypersensitive C-reactive protein (median 57.72 *vs*. 78.67 mg/L, *p* = 0.000). No significant differences were found in the positivity rates of tuberculous antibody (TbAb), purified protein derivative (PPD) skin test between the two groups (*p >*0.05). However, the positive rate of conventional acid-fast bacilli (AFB) smear was significantly higher in the PTB group than in the Non-PTB group (*p* = 0.03).

**Table 1:** Demographics and clinical characteristics of pulmonary tuberculosis (PTB) and non-pulmonary tuberculosis (non-PTB) patients (n=188).

Characteristics	Total (n= 188)	PTB (n= 116)	Non-PTB (n=72)	χ^2^/t/Z	*p-*value
Age (years). mean ± SD	62.9 ± 12.5	61.2 ± 13.4	65.3 ± 10.7	-17.2	0.000
Gender (Male/Female), n	132/56	88/28	44/28	4.62	0.032
Smoking history (≥ 5 years), n (%)	79(42.0)	51(44.0)	28(38.9)	0.470	0.493
Drinking history (≥ 3 years), n (%)	76(40.4)	49(42.2)	27(37.5)	0.415	0.520
Main clinical symptoms, n (%)					
Cough	167(88.8)	98(84.5)	69(95.8)	5.77	0.02
Expectoration	128(68.1)	69(59.5)	59(81.9)	10.32	0.001
Hemoptysis	57(30.3)	22(19.0)	35(48.6)	18.48	0.000
Dyspnea	23(12.2)	11(9.5)	12(16.7)	2.14	0.14
Fever	75(39.9)	40(34.5)	35(48.6)	3.69	0.054
Night sweats	33(17.6)	17(14.7)	16(22.2)	1.76	0.185
Chest tightness	58(30.9)	29(25.0)	29(40.3)	4.861	0.027
Chest pain	36(19.1)	16(13.8)	20(27.8)	5.61	0.018
Fatigue	44(23.4)	30(25.9)	14(19.4)	1.02	0.312
Underlying diseases, n (%)					
Pneumoconiosis	36(19.1)	22(19.0)	14(19.4)	0.01	0.935
Cardiovascular Disease	46(24.5)	29(25.0)	17(23.6)	0.05	0.83
Diabetes	9(4.8)	6(5.2)	3(4.2)	0.099	0.754
Chronic liver disease	11(5.9)	4(3.4)	7(9.7)	3.17	0.075
Chronic kidney diseases	1(0.5)	1(0.9)	0	0.624	0.430
Solid organ malignancy	12(6.4)	4(3.4) ^▲^	8(11.1) ^▲▲^	4.37	0.037
Hematologic tumor	1(0.5)	1(0.9) ^#^	0	0.624	0.430
Autoimmune disease	7(3.7)	4 (3.4) ^*^	3(4.2) ^**^	0.064	0.80
Chemotherapy	2(1.1)	1(0.9)	1(1.4)	0.117	0.732
Chronic pulmonary disease	51 (27.1)	3(2.6) ^Δ^	48(66.7) ^ΔΔ^	92.3	0.000
Laboratory tests, n (%)					
WBC (×10^9^/L; NR:3.5-9.5)	6.58(4.77,9.36)	5.54(4.23,7.75)	7.91(5.84,10.68)	-11.07	0.000
Lymphocyte (×10^9^/L; NR: 1.1-3.2)	1.43(1.03,2.10)	1.30(1.03,1.75)	1.60(1.15,2.68)	-3.24	0.001
Neutrophils (×10^9^/L; NR:1.8-7.5)	6.09(4.38,10.05)	5.54(4.28,7.75)	7.28(4.50,15.73)	-6.24	0.000
Hemoglobin (g/L; NR:115-175)	129.0(118.0,139.0)	130.0(115.0,141.0)	129.0(121.0,137.0)	-3.79	0.000
Albumin (g/L; NR:40-55)	38.39(35.12,41.42)	38.06(35.39,41.10)	38.79(35.09,42.99)	-3.59	0.000
ESR (mm/h; NR:0-20)	62.00(32.00,87.0)	59.00(34.00,87.0)	63.0(28.0,89.0)	-1.07	0.283
IL-6 (pg/ml; NR:0-6.6)	63.03(34.0,122.70)	62.53(36.4,96.5)	69.22(28.9,456.4)	-11.39	0.000
Hs-CRP (mg/L; NR:0-10)	63.29(37.82,91.03)	57.72(37.82,85.45)	78.67(32.02,100.50)	-7.70	0.000
LDH (U/L; NR:100-240)	190.5(168.4,230.3)	198.7(172.8,233.8)	179.0(162.8,213.2)	-20.21	0.000
PCT (ng/ml; NR: 0-0.5)	1.50(0.82,1.50)	0.11(0.03,0.48)	0.24(0.09,1.50)	-1.41	0.157
TbAb, % (Positive/Total)	51.1(96/188)	53.4(62/116)	47.2(34/72)	0.689	0.41
PPD, % (Positive/Total)	41.5(78/188)	45.7(53/116)	34.7(25/72)	2.20	0.138

In **Table 1**, continuous variables with a normal distribution, expressed as mean ± standard deviation (SD), were compared using the independent samples t-test, with the statistic denoted as *t*. Non-normally distributed continuous variables, expressed as median (interquartile range, 25th–75th percentiles), were compared using the Mann–Whitney U test, with the statistic denoted as *Z*. Categorical variables, expressed as frequencies and percentages [n (%)], were compared using the chi-square test, with the statistic denoted as χ².

^▲^ included one case of lung adenocarcinoma, one case of lung squamous cell carcinoma, one case of tongue cancer, one case of esophageal cancer, and one case of breast cancer;^▲▲^Included 5 cases of lung cancer, 1 case of esophageal cancer, 1 case of breast cancer, and 1 case of gallbladder cancer. ^#^ included one case with NK/T-cell lymphoma.

^*^ included 1 case of rheumatoid arthritis, 1 case of ankylosing spondylitis, 1 case of dermatomyositis, and 1 case of ankylosing spondylitis. ^**^ included 1 case of rheumatoid arthritis, 1 case of ankylosing spondylitis, and 1 case of autoimmune hemolytic anemia.

^Δ^ included 1 case of COPD, 1 case of bronchiectasis, and 1 case of bronchial asthma; ^ΔΔ^ included 22 cases of old pulmonary tuberculosis, 11 cases of bronchiectasis, 9 cases of COPD, 4 cases of pulmonary emphysema, 1 case of interstitial lung disease, and 1 case of pulmonary fibrosis.

### 3.2 Radiological and bronchoscopic findings

Chest CT and bronchoscopic features are summarized in **Table 2**. Although many typical imaging manifestations, such as consolidations, cavities, and pleural effusions, were similarly distributed between groups (*p*>0.05), diffuse millet-like nodules (17.2% *vs*. 5.6%, *p* = 0.02) and hilar/mediastinal lymph node enlargement (44.8% *vs*. 25.0%, *p* = 0.01) were significantly more common in the PTB group. Bronchoscopically, endobronchial nodules were observed exclusively in the PTB group (6.9% *vs*. 0%, *p* = 0.02), whereas purulent secretions were more frequently seen in the Non-PTB group (16.7% *vs*. 4.3%, *p* = 0.004).

**Table 2:** Chest CT and bronchoscopic presentation of enrolled patients (n=188).

Imaging findings	Total (n= 188)	PTB (n= 116)	Non-PTB (n=72)	χ^2^	*p-*value
Chest CT findings, n (%)					
Patch consolidations	112(59.6)	69(59.5)	43(59.7)	0.001	0.974
Ground glass opacities	13(6.9)	5(4.3)	8(11.1)	3.192	0.074
Cavity	71(37.8)	44(37.9)	27(37.5)	0.004	0.953
Tree bud sign	8(4.3)	3(2.6)	5(6.9)	2.07	0.15
Diffuse millet-like nodules	24(12.8)	20(17.2)	4(5.6)	5.45	0.02
Pleural effusions	64(34.0)	45(38.8)	19(26.4)	3.04	0.08
Hilar/mediastinal lymph node enlargement	70(37.2)	52(44.8)	18(25.0)	7.47	0.01
Bronchiectasis	40(21.3)	24(20.7)	16(22.2)	0.06	0.80
Site of lesions on chest CT, n (%)					
Upper lobe	71(37.8)	45(38.8)	26(36.1)	0.14	0.71
Middle lobe	21(11.2)	10(8.6)	11(15.3)	1.98	0.16
Lower lobe	57(30.3)	34(29.3)	23(31.9)	0.15	0.70
Multiple lobes (≥2)	39(20.7)	27(23.3)	12(16.7)	1.18	0.28
Bronchoscopic findings, n (%)					
Tracheobronchial ulcers	1(0.5)	1 (0.86)	0	0.62	0.43
Nodules	8(4.3)	8(6.90)	0	5.19	0.02
Necrosis	9(4.8)	8(6.90)	1(1.4)	2.96	0.09
Hyperemic or hypertrophic mucosa	60(31.9)	34(29.3)	26(36.1)	0.95	0.33
Purulent secretion	17(9.0)	5 (4.3)	12(16.7)	8.3	0.004
Nonspecific	93(49.5)	60(51.7)	33(45.8)	0.62	0.43

### 3.3 Comparison of the detection consistency of AFB, Culture, Xpert MTB/RIF and BALF mp-tNGS

As detailed in **Table 3**, the basis for CRS-based diagnosis among the 116 patients confirmed with PTB was distributed as follows: 39 cases (33.6%) were confirmed by positive BALF culture; 72 cases (62.1%) were diagnosed by histopathological examination (showing granulomatous inflammation with or without necrosis) combined with positive MTB-PCR on tissue samples, which were obtained via medical thoracoscopic pleural biopsy (35 cases), CT-guided percutaneous lung biopsy (17 cases), transbronchial cryobiopsy (11 cases), transbronchial needle aspiration (7 cases), and transbronchoscopic biopsy (2 cases); an additional 5 cases (4.3%) were clinically diagnosed based on highly suggestive clinical and radiological presentations of PTB along with a favorable response to standard anti-tuberculosis treatment, in the absence of microbiological or pathological confirmation.

**Table 3:** The final diagnostic methods of enrolled patients (n=188).

Diagnostic methods	Total (n= 188)	PTB (n= 116)	Non-PTB (n=72)
Culture	90(47.9)	39(33.6)	51(70.8)^Δ^
Histopathology+PCR			
Medical thoracoscopy	45(23.9)	35(30.2)**^▲^**	10(13.9) **^#^**
CT-guided PLB	23(12.2)	17(14.7) **^▲^**	6(8.3)**^※^**
TBB	12(6.4)	11(9.5) **^▲^**	1(1.4)**^☆^**
TBNA	11(5.9)	7(6.0) **^▲^**	4(15.3)**^★^**
TBCB	2(1.1)	2(1.7) **^▲^**	0
Favorable response to anti-TB treatment	5(2.7)	5(4.3)	0

**^▲^** Histopathology from all patients indicated granulomatous inflammation with or without necrosis, and all were positive for *Mycobacterium tuberculosis* PCR; ^Δ^included 19 cases of chronic pulmonary aspergillosis, 11 cases of necrotizing pneumonia/lung abscess (caused by *Klebsiella pneumoniae* [n=6] and *Staphylococcus aureus* [n=5]), 10 cases of chronic *Pseudomonas aeruginosa* infection, 5 cases of pulmonary cryptococcosis, 5 cases of nontuberculous mycobacterial pulmonary disease (NTM-PD), and 1 case of nocardiosis; **^#^**Included 4 cases of nonspecific pleuritis, 3 cases of lung adenocarcinoma, 2 cases of paragonimiasis, and 1 case of empyema; **^※^** Included 2 cases of nonspecific chronic inflammation, 1 cases of organizing pneumonia, 1 cases of lung abscess, 1 cases of NTM, and 1 cases of granulomatous disease; **^☆^**1 case of *Proteus mirabilis* infection; **^★^**Included 2 cases of sarcoidosis, 1 case of lymph node metastasis from lung cancer, and 1 case of lymphadenitis.

**Figure 2:**
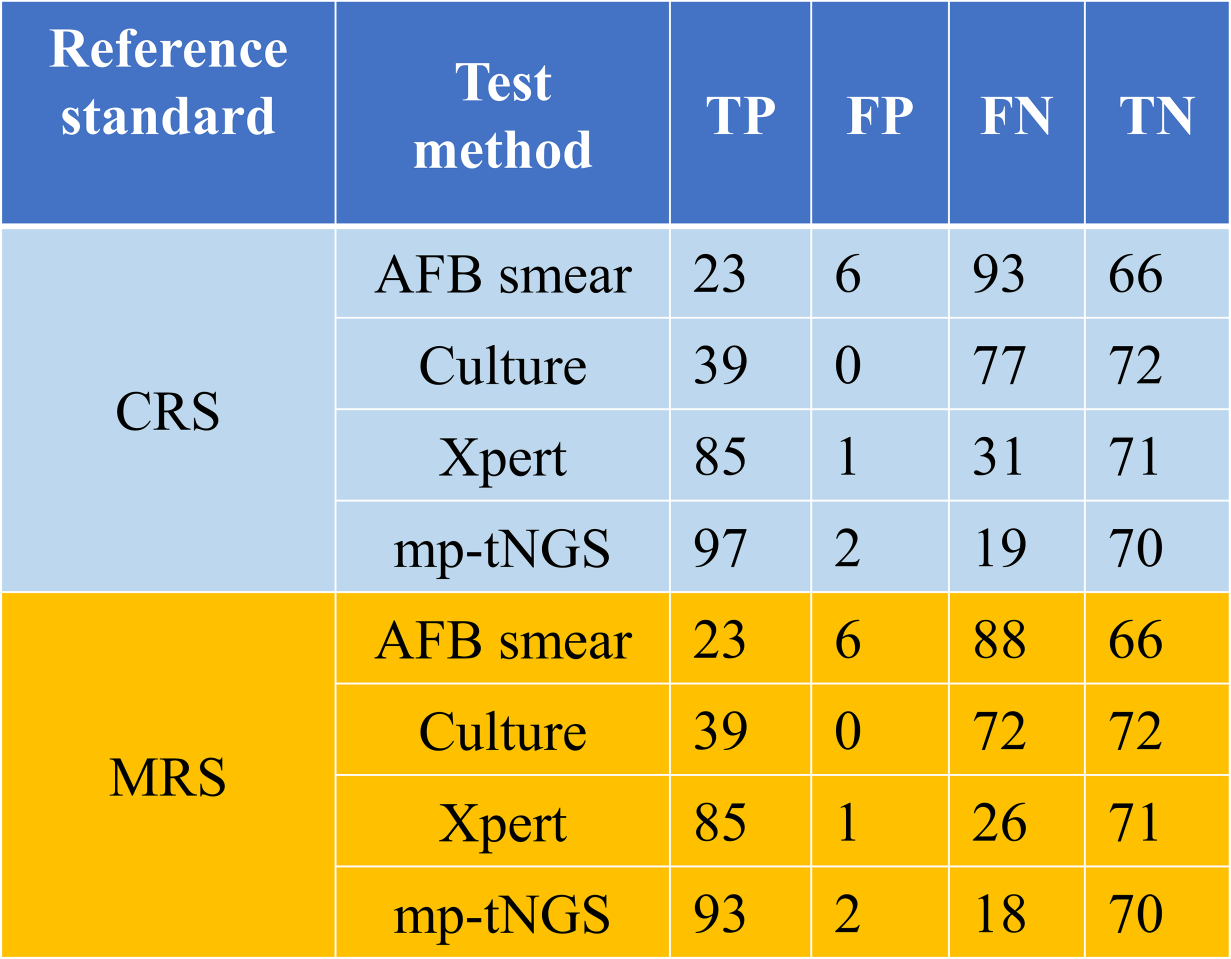
Contingency table results of four diagnostic tests (AFB smear, culture, Xpert MTB/RIF, and mp-tNGS) for tuberculosis, using composite reference standard (CRS) and microbiological reference standard (MRS) as reference standards. TP, true positive; FP, false positive; FN, false negative; TN, true negative.

**Figure 3:**
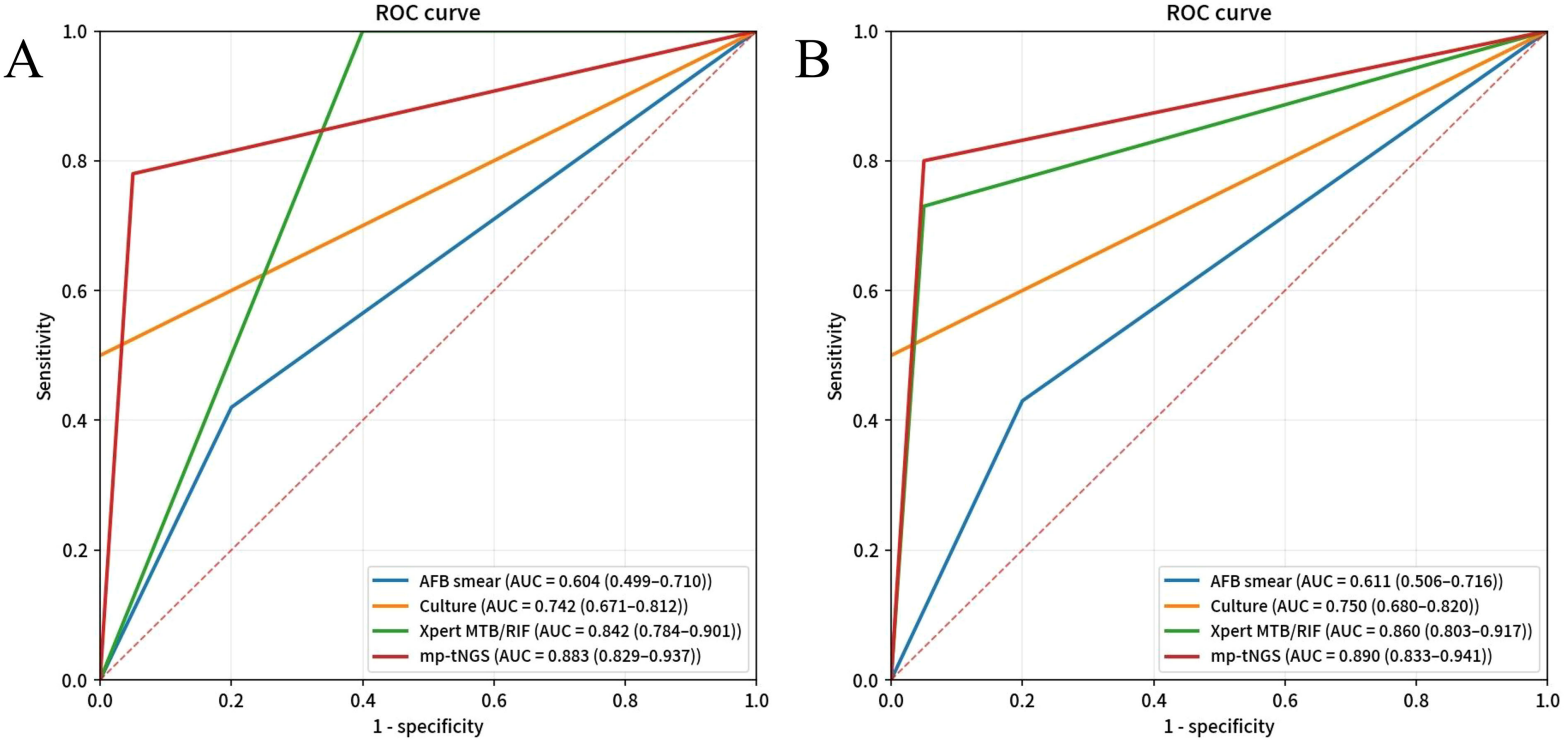
Receiver operating characteristic (ROC) curves of AFB smear, culture, Xpert MTB/RIF, and mp−tNGS for MTB detection. **(A)** Based on the composite clinical reference standard (CRS; n = 188). **(B)** Based on the microbiological reference standard (MRS; n = 183).

Among the 72 non-PTB patients, 51 (70.8%) had positive BALF cultures for other pathogens, including *Aspergillus* spp. (19 cases), *Klebsiella pneumoniae* (6 cases), *Staphylococcus aureus* (5 cases), *Pseudomonas aeruginosa* (10 cases), nontuberculous mycobacteria (5 cases), and *Cryptococcus* spp. (5 cases), with some cases involving mixed infections. The remaining 21 patients were diagnosed by pathological examination of biopsies obtained through various methods, including: nonspecific inflammation or pleuritis (6 cases), lung adenocarcinoma (3 cases), paragonimiasis (2 cases), empyema (1 case), sarcoidosis (2 cases), lymph node metastasis from lung cancer (1 case), lymphadenitis (1 case), *Proteus mirabilis* infection (1 case), organizing pneumonia (1 case), lung abscess (1 case), and other granulomatous diseases (2 cases). In the present study, the CRS served as the primary reference standard for evaluating diagnostic consistency, while the MRS was used for secondary subgroup validation. As shown in **Figure 2**, diagnostic four-fold table revealed the following: on the basis of CRS diagnosis, both AFB smear and mycobacterial culture demonstrated poor concordance, with κ values (± standard error) of 0.094 ± 0.041 (*p* = 0.03) and 0.28 ± 0.44 (*p* < 0.001), respectively. AFB smear yielded only 23 true positives, along with 6 false positives (all attributable to nontuberculous mycobacteria) and 93 false negatives. MTB culture identified 39 true positives with no false positives among non-PTB cases, but missed 77 PTB cases. Xpert MTB/RIF showed good agreement (κ = 0.658 ± 0.051, *p* < 0.001), detecting 85 true positives, with only one false positive and 31 missed PTB cases. In contrast, mp-tNGS exhibited the strongest agreement (κ ± SE = 0.774 ± 0.046, *p* < 0.001), identifying the highest number of true positives (97 cases) and presenting only 2 false positives and 19 false negatives. Correspondingly, receiver operating characteristic (ROC) analysis indicated that the area under the curve (AUC) (AUC± SE) was 0.604 ± 0.054 (95% CI: 0.499–0.710, *p=*0.075) for AFB smear, 0.742 ± 0.036 (95% CI: 0.671–0.812, *p* < 0.001) for culture, 0.842 ± 0.030 (95% CI: 0.784–0.901, *p* < 0.001) for Xpert MTB/RIF, and highest for mp-tNGS at 0.883 ± 0.028 (95% CI: 0.829–0.937, *p* < 0.001). When the MRS was applied as the reference standard, AFB smear and culture again yielded low κ values (0.103 ± 0.043, *p* = 0.025 and 0.299 ± 0.046, *p* < 0.001, respectively). Xpert MTB/RIF achieved a κ of 0.709 ± 0.050 (*p* < 0.001), with 85 true positives, one false positive, and 26 missed PTB cases. mp-tNGS gave the highest κ (0.780 ± 0.046, *p* < 0.001), correctly identifying 93 true positives, with only 2 false positives and 18 false negatives. ROC analysis under the MRS yielded AUCs of 0.611 ± 0.054 (95% CI: 0.506–0.716, *p* = 0.059) for AFB smear, 0.750 ± 0.036 (95% CI: 0.680–0.820, *p* < 0.001) for culture, 0.860 ± 0.029 (95% CI: 0.803–0.917, *p* < 0.001) for Xpert MTB/RIF, and 0.890 ± 0.027 (95% CI: 0.833–0.941, *p* < 0.001) for mp-tNGS, again highest for mp-tNGS. The ROC curves of the four diagnostic tests under both CRS and MRS are presented in **Figure 3**.

### 3.4 Comparison of the detection performance of AFB, Culture, Xpert MTB/RIF and BALF mp-tNGS

Based on the composite clinical reference standard (CRS, n = 188), the sensitivity of AFB smear for MTB detection was low at 19.8% (95% CI: 13.0–28.3), with a specificity of 91.7% (95% CI: 82.7–96.9). Mycobacterial culture exhibited a sensitivity of 33.6% (95% CI: 25.1–43.0) and the highest specificity of 100% (95% CI: 95.0–100.0). The Xpert MTB/RIF assay showed a moderate sensitivity of 73.3% (95% CI: 64.3–81.1) while maintaining high specificity of 98.6% (95% CI: 92.5–99.9). In contrast, mp-tNGS demonstrated a significantly superior sensitivity of 83.6% (95% CI: 78.6–91.9) with a specificity of 97.2% (95% CI: 90.3–99.6). Accordingly, mp-tNGS achieved the highest diagnostic accuracy (88.8%, 95% CI: 83.4–92.9) and the largest area under the ROC curve (AUC 0.883 ± 0.028, 95% CI: 0.829–0.937, *p* < 0.001), outperforming AFB smear (AUC 0.604 ± 0.054, 95% CI: 0.499–0.710, *p* = 0.075), culture (AUC 0.742 ± 0.036, 95% CI: 0.671–0.812, *p* < 0.001), and Xpert MTB/RIF (AUC 0.842 ± 0.030, 95% CI: 0.784–0.901, *p* < 0.001). Regarding turnaround time, AFB smear was the fastest (mean 1.1 ± 0.13 h), followed by Xpert MTB/RIF (2.13 ± 0.20 h) and mp-tNGS (12.10 ± 2.49 h), whereas culture required the longest processing time (339.80 ± 139.5 h). Details are shown in **Table 4**.

**Table 4:** Comparison of diagnostic performance for MTB detection in the CRS confirmed subgroup (n=188).

Test method	Sensitivity, % (95% CI)	Specificity, % (95% CI)	PPV, % (95% CI)	NPV, % (95% CI)	Diagnostic accuracy, % (95% CI)	AUC (95% CI)	TAT, hours (Mean ± SD)
AFB smear	19.8(13.0-28.3)	91.7(82.7-96.9)	79.3(62.1-89.9)	41.5(38.8-44.3)	47.3(40.0-54.7)	0.604 (0.499–0.710)	1.1 ± 0.13
Culture	33.6(25.1-43.0)	100.0(95.0-100.0)	100.0	48.3(45.1-51.6)	59.0(51.7-66.1)	0.742 (0.671–0.812)	339.80 ± 139.5
Xpert MTB/RIF	73.3(64.3-81.1)	98.6(92.5-99.9)	98.8(92.4-99.8)	69.6(62.9-75.6)	83.0(76.8-88.1)	0.842 (0.784–0.901)	2.13 ± 0.20
mp-tNGS	83.6(78.6-91.9)	97.2(90.3-99.6)	98.0(93.1-99.5)	78.6(70.9-84.8)	88.8(83.4-92.9)	0.883 (0.829–0.937)	11.50 ± 2.49

PPV, positive predictive value; NPV, negative predictive value; AUC, area under the curve; TAT, turnaround time; CI, confidence interval; SD, standard deviation.

**Figure 4:**
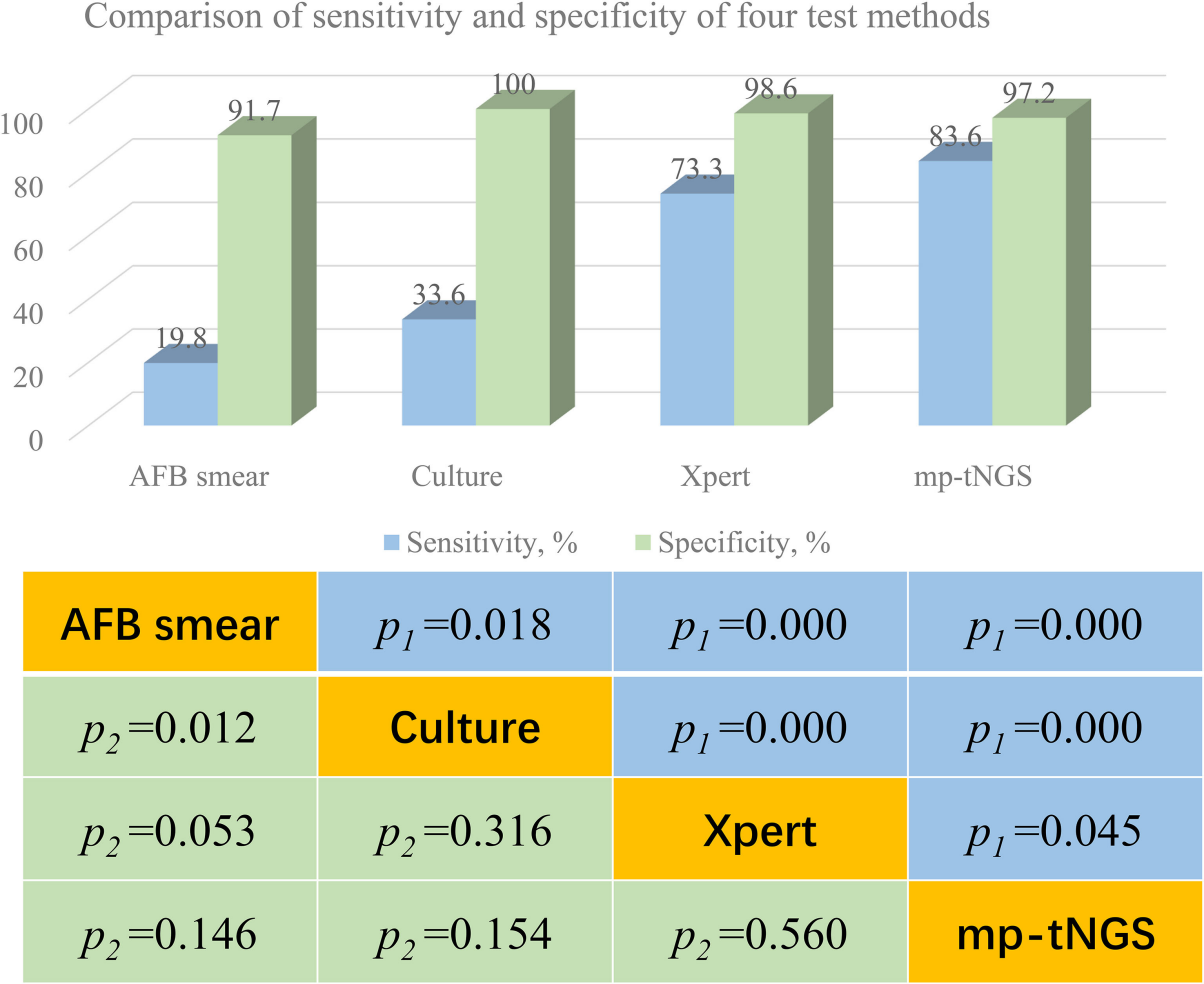
Diagnostic sensitivity and specificity were compared across different assays for Mycobacterium tuberculosis (MTB) detection. p1 and p2 represent the p values for comparisons of sensitivity and specificity, respectively. Abbreviations: AFB, acid-fast bacilli; mp- tNGS, multiplex PCR-based targeted next-generation sequencing.

As illustrated in **Figure 4**, comparative analysis of sensitivity revealed a hierarchical performance among the four assays. The sensitivity of mp-tNGS (83.6%, 97/116) was significantly higher than that of Xpert MTB/RIF (73.3%, 85/116), with a difference of 10.3 percentage points (95% CI: 1.2–19.4%; McNemar’s test, χ² = 4.50, *p* = 0.045). Similarly, mp-tNGS demonstrated superior sensitivity compared to culture (difference: 50.0 percentage points; 95% CI: 39.8–60.2%; *p* < 0.001) and AFB smear (difference: 63.8 percentage points; 95% CI: 52.9–74.7%; *p* < 0.001). Culture also outperformed AFB smear (difference: 13.8 percentage points; 95% CI: 3.0–24.6%; *p* = 0.018), and Xpert MTB/RIF showed higher sensitivity than culture (difference: 39.7 percentage points; 95% CI: 29.2–50.2%; *p* < 0.001). In terms of specificity, no significant differences were observed among mp-tNGS (97.2%), culture (100%), and Xpert MTB/RIF (98.6%) when compared pairwise (all *p* > 0.05), whereas AFB smear (91.7%) had lower specificity than culture (difference: -8.3 percentage points; 95% CI: -15.3 to -1.3%; *p* < 0.05).

To validate the robustness of our primary CRS−based estimates, we performed a subgroup analysis restricted to microbiologically or histopathologically confirmed PTB cases (MRS, n = 111), excluding the five clinically diagnosed patients. Among the 116 PTB patients, 111 (95.7%) had definitive microbiological confirmation by positive BALF culture (n = 39) or histopathology with positive MTB PCR (n = 72). In this MRS-confirmed cohort, as shown in **Table 5**, mp-tNGS demonstrated a sensitivity of 83.8% (93/111; 95% CI: 75.5–90.1%), a specificity of 97.2% (95% CI: 90.3–99.6%), a diagnostic accuracy of 89.1% (95% CI: 83.6–93.1%), and an AUC of 0.890 ± 0.027 (95% CI: 0.833–0.941, *p* < 0.001). These values were nearly identical to those observed in the full CRS-based cohort (sensitivity 83.6%, specificity 97.2%, AUC 0.883 ± 0.028). The strong concordance between CRS- and MRS-based estimates indicates that the inclusion of clinically diagnosed patients did not materially affect the diagnostic performance measures. Notably, the sensitivity of mp-tNGS in the MRS-confirmed cohort remained significantly higher than that of Xpert MTB/RIF (76.6%, 95% CI: 67.6–84.0%) and culture (35.1%, 95% CI: 26.3–44.8%), further confirming the superior detection capability of mp-tNGS even under the MRS.

**Table 5:** Comparison of diagnostic performance for MTB detection in the MRS confirmed subgroup (n = 183).

Test method	Sensitivity, % (95% CI)	Specificity, % (95% CI)	PPV, % (95% CI)	NPV, % (95% CI)	Diagnostic accuracy, % (95% CI)	AUC (95% CI)	TAT, hours (Mean ± SD)
AFB smear	20.7 (13.6–29.5)	91.7 (82.7–96.9)	79.3(62.1-89.9)	42.9(39.6-46.3)	48.6 (41.3–56.0)	0.611 (50.6–71.6)	1.1 ± 0.13
Culture	35.1 (26.3–44.8)	100.0(95.0-100.0)	100.0	50.0(46.4–53.6)	60.7 (53.3–67.7)	0.750 (68.0–82.0)	339.80 ± 139.5
Xpert MTB/RIF	76.6 (67.6–84.0)	98.6(92.5-99.9)	98.8(92.4-99.8)	73.2(66.0–79.4)	85.2 (79.2–90.0)	0.860(80.3–91.7)	2.13 ± 0.20
mp-tNGS	83.8 (75.5–90.1)	97.2(90.3-99.6)	97.9 (92.7–99.5)	79.5(71.5–85.8)	89.1 (83.6–93.1)	0.890 (83.3–94.1)	11.50 ± 2.49

This table is based on the microbiological reference standard (MRS) subgroup, which included 111 patients with microbiologically or histopathologically confirmed PTB (positive culture or histopathology with MTB PCR) and 72 non PTB patients. The five clinically diagnosed PTB patients (without microbiological proof) were excluded. AUC values are unchanged from the primary analysis as exclusion of the five patients did not materially affect ROC curves.

To further evaluate the diagnostic performance of mp-tNGS in paucibacillary populations, we performed stratified analyses based on smear status (AFB smear-positive *vs*. smear-negative) and culture status (culture-positive *vs*. culture-negative) in the 116 PTB patients. The results are summarized in **Table 6**. Among the 116 PTB patients, 23 (19.8%) were smear-positive and 93 (80.2%) were smear-negative. mp-tNGS achieved 100% sensitivity (23/23) in smear-positive PTB and 77.4% sensitivity (72/93; 95% CI: 67.6–85.3%) in smear-negative PTB. In comparison, Xpert MTB/RIF showed 100% sensitivity in smear-positive cases and 66.7% sensitivity (62/93; 95% CI: 56.2–76.1%) in smear-negative cases, while culture detected only 1.1% (1/93) of smear-negative PTB cases. By culture status, 39 (33.6%) PTB patients were culture-positive and 77 (66.4%) were culture-negative. mp-tNGS demonstrated 100% sensitivity (39/39) in culture-positive PTB and 74.0% sensitivity (57/77; 95% CI: 62.8–83.4%) in culture-negative PTB. Xpert MTB/RIF showed 100% sensitivity in culture-positive cases and 59.7% sensitivity (46/77; 95% CI: 48.0–70.6%) in culture-negative cases. These findings confirm that mp-tNGS maintains robust diagnostic performance even in smear-negative and culture-negative paucibacillary populations, with specificity remaining at 97.2% across all subgroups.

**Table 6:** Sensitivity of mp−tNGS and Xpert MTB/RIF for PTB detection stratified by smear and culture status (based on CRS, n = 116).

Subgroup	n (% of PTB)	mp-tNGS sensitivity, % (95% CI)	Xpert MTB/RIF sensitivity, % (95% CI)	Culture sensitivity, % (95% CI)
By smear status				
Smear-positive	23 (19.8%)	100% (23/23)	100% (23/23)	100% (23/23)
Smear-negative	93 (80.2%)	77.4% (72/93; 67.6–85.3%)	66.7% (62/93; 56.2–76.1%)	1.1% (1/93; 0.03–5.9%)
By culture status				
Culture-positive	39 (33.6%)	100% (39/39)	100% (39/39)	100% (39/39)
Culture-negative	77 (66.4%)	74.0% (57/77; 62.8–83.4%)	59.7% (46/77; 48.0–70.6%)	—

### 3.5 Comparison of the performance of culture, Xpert MTB/RIF, and BALF mp-tNGS for drug resistance detection

The comparative performance of BALF mp-tNGS, phenotypic drug susceptibility testing (pDST), and Xpert MTB/RIF in detecting drug resistance among 35 PTB patients is detailed in **Table 7**. Mp-tNGS identified genotypic resistance to rifampicin (RIF) in 8 cases (all with *rpoB* S450L mutation) and to isoniazid (INH) in 6 cases (all with *katG* S315T mutation). Using pDST as the reference standard, the sensitivity and specificity of tNGS for RIF resistance were both 100%. For INH resistance, tNGS showed a sensitivity of 75% (6/8) and a specificity of 100% (27/27), as two pDST-confirmed INH-resistant cases (Case 2 and Case 5) lacked *katG* mutations detectable by tNGS. Xpert MTB/RIF detected RIF resistance in 6 of the 8 pDST-confirmed RIF-resistant cases (sensitivity 75.0%), and among the remaining 27 cases, one false positive result due to INH resistance was observed, yielding a specificity of 96.3%. Notably, tNGS successfully identified 5 cases (Cases 1, 4, 6, 10, and implied in Case 3 based on pDST) carrying concurrent *rpoB* and *katG* mutations, signaling probable multidrug-resistant (MDR-TB) profiles. pDST further revealed broader resistance patterns in several of these cases, including resistance to fluoroquinolones (e.g., ofloxacin, moxifloxacin) and second-line injectables (e.g., streptomycin, amikacin). One case of ethambutol (EMB) resistance detected by pDST (Case 6) was not identified by tNGS, reflecting a limitation in its genetic coverage. In summary, while tNGS provides rapid genotypic resistance screening with high specificity and is particularly useful for early MDR-TB suspicion, its sensitivity for INH resistance remains suboptimal. Given the limited sample size (n=35), this resistance findings should be interpreted as exploratory and require validation in larger prospective cohorts. Xpert MTB/RIF offers a reliable and rapid screen for RIF resistance, though it may miss a small proportion of resistant cases. Phenotypic DST remains essential for confirming resistance, detecting resistance to drugs not covered by genetic assays, and guiding definitive treatment.

**Table 7:** Comparison of the efficacy of culture, Xpert MTB/RIF, and BALF mp-tNGS for drug resistance detection in PTB patients (n=35).

Cases	Genotypic resistance (tNGS)	Resistance gene & mutation	pDST	Xpert MTB/RIF
Case 1	RIF; INH	*rpoB* S450L; *katG* S315T	RIF, INH, ofloxacin, moxifloxacin	RIF
Case 2	RIF	*rpoB* S450L	RIF	RIF
Case 3	INH	*katG* S315T	INH, streptomycin	RIF
Case 4	RIF; INH	*rpoB* S450L; *katG* S315T	RIF, INH, ofloxacin, Moxifloxacin, amikacin	RIF
Case 5	RIF	*rpoB* S450L	RIF, INH	
Case 6	RIF; INH	*rpoB* S450L; *katG* S315T	RIF, INH, EMB, ofloxacin, moxifloxacin, streptomycin	RIF
Case 7	RIF	*rpoB* S450L	RIF	RIF
Case 8	RIF	*rpoB* S450L	RIF	
Case 9	INH	*katG* S315T	INH	
Case 10	RIF; INH	*rpoB* S450L; *katG* S315T	RIF, INH, ofloxacin, moxifloxacin	RIF

## 4 Discussions

This prospective diagnostic performance study systematically demonstrates the superior capability of mp-tNGS for detecting MTB in BALF. Compared to conventional methods—AFB smear, culture, and Xpert MTB/RIF—mp-tNGS achieved the highest sensitivity (83.6% *vs*. 19.8–73.3%), diagnostic accuracy (88.8%), and agreement with a composite clinical reference standard (κ = 0.868). Furthermore, mp-tNGS enabled simultaneous pathogen identification and rapid genotypic screening for common anti-tuberculosis drug resistance within approximately 12 hours, providing a powerful tool for early clinical decision-making.

The diagnosis of smear-negative and paucibacillary pulmonary tuberculosis (PTB) remains a significant clinical challenge (Wang et al., 2014; Tang et al., 2023). Conventional methods are limited: AFB smear has low sensitivity and cannot differentiate MTB from nontuberculous mycobacteria (NTM) (2, 3); liquid culture, while the traditional gold standard, requires weeks for results (4); and although widely adopted, Xpert MTB/RIF exhibits limited sensitivity in paucibacillary specimens like BALF (73.3% in this study) (Maynard-Smith et al., 2014; Chakravorty et al., 2017; Tang et al., 2023). The diagnostic challenge of paucibacillary PTB has spurred the evaluation of various NGS technologies. Studies such as those by Lin et al. (2025) and Yang et al (Yang Z. et al., 2025). have demonstrated the utility of mNGS in detecting MTB and co-infections across diverse sample types, including lymph node tissue and BALF. Furthermore, tNGS approaches, such as targeted nanopore sequencing and multiplex PCR-based panels, have consistently shown superior sensitivity (77.7%-91.6%) over conventional methods like smear microscopy, culture, and Xpert MTB/RIF in respiratory specimens (Sun et al., 2023; Qianfang et al., 2025; Wu et al., 2025). In our cohort, mp-tNGS detected 97 out of 116 confirmed PTB cases, including 34 missed by Xpert. This aligns with recent studies evaluating tNGS technologies. For instance, Sun et al. (2023) reported a sensitivity of 89.6% for targeted nanopore sequencing in BALF, significantly outperforming Xpert (56.2%) and culture (39.6%) (Sun et al., 2023). Similarly, Wu et al. (2025) demonstrated that tNGS detected MTB in 111 out of 158 BALF samples, far exceeding culture (29) and Xpert (70) yields, and identified 45% of clinically diagnosed cases as positive (Wu et al., 2025). These findings collectively indicate that tNGS, through efficient capture and deep sequencing of multi-copy targets (e.g., IS6110), significantly enhances detection in low-biomass samples, potentially addressing the critical diagnostic gap for smear-negative PTB. Stratified analysis in our cohort further quantified this advantage: mp-tNGS achieved a sensitivity of 77.4% (72/93) in smear-negative PTB and 74.0% (57/77) in culture-negative PTB, outperforming Xpert MTB/RIF by 10.7 and 14.3 percentage points, respectively. These findings align with a recent evaluation of targeted nanopore sequencing, which reported detection rates of 93.8% for smear-negative and 89.1% for culture-negative tuberculosis patients (Yang C. et al., 2025), confirming that tNGS-based approaches are particularly well-suited for low-bacterial-load specimens where conventional methods fall short.

Beyond pathogen identification, a key strength of mp-tNGS is its integrated drug resistance gene detection. In our resistance testing subset (n=35), mp-tNGS demonstrated 100% sensitivity and specificity for rifampicin resistance (all *rpoB* S450L mutations) and 75% sensitivity with 100% specificity for isoniazid resistance (*katG* S315T mutations), successfully flagging five probable multidrug-resistant (MDR) TB cases. In contrast, Xpert MTB/RIF missed two rifampicin-resistant cases in this study and provides no information on resistance to isoniazid or other drugs (Günther et al., 2022). This echoes the evaluation by Ou et al. (2025) of the “TB Pro” assay, where tNGS showed high concordance with phenotypic drug susceptibility testing (pDST) and whole-genome sequencing (WGS) for predicting resistance to rifampicin, isoniazid, fluoroquinolones, and others (sensitivity 74.3–94.4%, specificity >98%) (Ou et al., 2025). Although mp-tNGS coverage of certain resistance mechanisms (e.g., non-*katG* S315T INH resistance) is not exhaustive, limiting sensitivity, its ability to provide a multi-drug resistance profile in a single assay holds irreplaceable clinical value for early suspicion of MDR-TB and guiding empirical therapy adjustment, potentially avoiding treatment delays associated with lengthy pDST turnaround times (Houben and Dodd, 2016; Gong and Wu, 2021).

The technical advantage of mp-tNGS stems from the combination of “targeted enrichment” and “high-throughput sequencing.” Compared to metagenomic NGS (mNGS), pre-amplification with specific primers reduces host nucleic acid background, increases detection sensitivity for target pathogens, and significantly lowers sequencing data volume and cost (Miller et al., 2019; Yin et al., 2024). The panel used in this study covers 198 common respiratory pathogens, granting it potential for the differential diagnosis of complex pulmonary infections (e.g., mixed infections with NTM, fungi, or bacteria) (Yin et al., 2024; Wang et al., 2025b; Wang et al., 2025a). From a clinical workflow perspective, the approximately 12-hour turnaround time for mp-tNGS, while longer than Xpert (~2 hours), is vastly superior to culture (weeks), striking a favorable balance between speed and comprehensiveness. Therefore, this technology is particularly suited for tertiary hospitals or regional reference laboratories with molecular diagnostic infrastructure, serving as a complementary or reflex test for cases with negative Xpert results but high clinical suspicion of PTB, or for suspected drug-resistant/mixed infections (Expert consensus on the application and practice of targeted next-generation sequencing in infectious diseases, 2024; Yin et al., 2024). The Non−PTB group had higher IL−6 and hs−CRP (*p* = 0.000), consistent with acute infections (bacterial pneumonia, lung abscess, CPA) rather than the indolent inflammation of PTB (Chakravorty et al., 2017). The lower albumin in PTB reflects its chronic catabolic state (2); the small absolute difference (0.73 g/L) is explained by early−stage disease (80.2% smear−negative). None of these markers individually discriminates PTB with high accuracy.

Two false-positive mp-tNGS results were observed. Case 1: a 54-year-old male with *Mycobacterium avium* complex pulmonary disease (confirmed by culture). Mp-tNGS detected 8 MTB reads (RPhK = 2.3); all other tests negative. The signal likely reflects cross-reactivity between IS6110 primers and homologous sequences in *M. avium* (Dziadek et al., 2001a; Dziadek et al., 2001b). Case 2: a 67-year-old female with chronic pulmonary aspergillosis (confirmed by BALF galactomannan and culture). Mp-tNGS detected 5 MTB reads (RPhK = 1.5); all other tests negative, no TB after 6-month follow-up. This likely represents trace laboratory contamination. These cases underscore the need to interpret low-level positive results (5–<20 reads) with confirmatory testing and clinical correlation. These two false-positive cases highlight an inherent trade-off of PCR-based tNGS: high sensitivity for paucibacillary MTB comes with a small risk of false positives due to NTM cross-reactivity or trace contamination. Hence, low-level positive results should be interpreted alongside clinical, radiological, and confirmatory findings.

Although a formal cost−effectiveness analysis was beyond this study’s scope, practical implementation considerations are key for real−world adoption. First, per−sample reagent and sequencing costs for mp-tNGS are approximately 80-100USD in our setting, *versus*10–15 for Xpert MTB/RIF and $15–20 for liquid culture. Despite higher upfront cost, mp−tNGS may offer favorable health economic value by averting misdiagnoses, reducing empirical therapy, shortening hospital stays, and enabling earlier appropriate therapy (de Nooy et al., 2026). Second, mp−tNGS requires a molecular diagnostic laboratory with a real−time PCR cycler, a benchtop sequencer, and bioinformatics capacity, as outlined in the 2024 Chinese expert consensus (Expert consensus on the application and practice of targeted next-generation sequencing in infectious diseases, 2024). Successful implementation in a high−burden, resource−limited setting using a centralized reference laboratory model has been demonstrated in Namibia (de Araujo et al., 2023; Gopalaswamy et al., 2026). Third, the 12−hour turnaround time (TAT) was achieved with batch processing (20–30 samples/run); stat processing may extend TAT to 24–48 hours, but regular daily batching maintains clinically actionable TATs with cost efficiency. Fourth, while not suited for point−of−care use, a hub−and−spoke model (sample transport to a central lab) has been successful in high−burden settings. Centralized tNGS was cost−saving in South Africa, shortening TAT by 42.3 days and reducing infectious time by 97 years (de Nooy et al., 2026); decentralized tNGS remained cost−effective under current assumptions (de Nooy et al., 2026). Emerging compact tNGS platforms requiring minimal biocontainment may further lower barriers (Gopalaswamy et al., 2026). Thus, mp−tNGS is a realistic tool for centralized TB reference laboratories in high−burden countries, serving as a reflex test for smear−negative or Xpert−negative patients with high clinical suspicion.

mp-tNGS differentiates MTB from NTM via species-specific sequences. In our cohort, all 5 NTM-PD cases (2 *M. avium*, 2 *M. kansasii*, 1 *M. abscessus*) were correctly identified at species level; none had MTB reads meeting positivity threshold (≥5 reads, RPhK ≥1.5). One *M. avium* case generated 8 MTB reads – known IS6110 cross-reactivity (Mulcahy et al., 1996; Dziadek et al., 2001a). The 2024 Chinese expert consensus recommends tNGS covering both MTBC and pathogenic NTM for suspected MTB or AFB-positive cases needing NTM differentiation (Expert consensus on the application and practice of targeted next-generation sequencing in infectious diseases, 2024), which our panel follows. Ou et al. (2025) identified 10 MTBC and 39 NTM species (LoD: 3.0 CFU/mL for MTB, 1.4–16.2 for NTM) (Ou et al., 2025); another study reported 100% sensitivity and 97.5% specificity for NTM-PD (Tao et al., 2024). Deep sequencing can resolve NTM mixtures and MTB-NTM co-infections (Opperman et al., 2024). Thus, mp-tNGS reliably distinguishes MTB from NTM, with rare cross-reactivity confined to specific NTM at low read thresholds.

Several limitations of this study should be acknowledged. First, as with all tuberculosis diagnostic accuracy studies, the absence of a perfect gold standard poses a methodological challenge. Our primary analysis employed a composite CRS integrating microbiology, histopathology, imaging, and treatment response. While the CRS is widely accepted, it carries potential risks of incorporation and verification bias. To mitigate this concern, we performed a secondary subgroup analysis restricted to microbiologically confirmed cases (MRS, n = 111), which yielded nearly identical sensitivity (83.8% vs. 83.6%) and specificity (97.2% vs. 97.2%) to the primary CRS−based estimates, confirming the robustness of our findings. Nevertheless, residual bias cannot be entirely excluded. Future studies using Bayesian latent class analysis (LCA) could provide less biased accuracy estimates for mp−tNGS (Keter et al., 2023; Keter et al., 2024). Second, as a single-center study, the sample size (n=188) is relatively limited. Future validation in larger, multicenter cohorts is warranted to confirm generalizability, especially among special populations such as people living with HIV or children. Third, while the mp-tNGS panel covers common resistance mutations, it does not encompass all gene variants potentially associated with resistance in the WHO catalogue (e.g., *ahpC* promoter mutations), which may explain its suboptimal sensitivity for INH resistance (Ko et al., 2019; Ou et al., 2025). Our assessment of mp−tNGS for drug resistance profiling is based on a limited subset (n=35). While excellent for rifampicin, the 75% sensitivity for isoniazid resistance—likely due to the sample size and uncovered mechanisms beyond katG S315T—should be considered preliminary. Phenotypic DST remains essential for confirmation and detecting resistance to uncovered drugs. These results highlight the need for larger, prospective studies to validate mp−tNGS for comprehensive genotypic resistance screening in clinical practice. Fourth, a cost-effectiveness analysis of mp-tNGS was not conducted. Although its reagent and sequencing costs are higher than Xpert, its overall health economic value—considering the benefits of preventing misdiagnosis, shortening time to diagnosis, and enabling early guided therapy—may be significant and merits future assessment (Policy Statement: Automated Real-Time Nucleic Acid Amplification Technology for Rapid and Simultaneous Detection of Tuberculosis and Rifampicin Resistance: Xpert Mtb/Rif System, 2011). Finally, further research is needed to better integrate mp-tNGS results with clinical decision thresholds (e.g., differentiating colonization from infection). The work by Chen et al. (2024), combining tNGS with machine learning to establish diagnostic thresholds for plasma and cerebrospinal fluid samples, offers a novel approach for optimizing mp-tNGS data interpretation in the future (Chen S. et al., 2024).

In conclusion, this study confirms a good diagnostic performance of mp-tNGS for PTB, especially smear-negative and paucibacillary disease, in BALF samples. Our findings suggest that mp-tNGS can provide clinicians with early and robust evidence to inform decisions, potentially improving the management pathway and prognosis for PTB patients. Future efforts should prioritize multicenter validation and assessment of its utility in TB surveillance programs.

## Data Availability

The raw data supporting the conclusions of this article will be made available by the authors, without undue reservation.

## Glossary

PCR: Polymerase chain reaction
NGS: Next-generation sequencing
MTB: Mycobacterium tuberculosis
BALF: Bronchoalveolar lavage fluid
PTB: Pulmonary tuberculosis
AFB: Acid-fast bacilli
RIF: Rifampicin
pDST: Phenotypic drug susceptibility testing
WHO: World Health Organization
TB: Tuberculosis
NTM: Nontuberculous mycobacteria
TST: Tuberculin skin test
LTBI: Latent tuberculosis infection
IGRA: Interferon-gamma release assay
mNGS: Metagenomic next-generation sequencing
tNGS: Targeted next-generation sequencing
mp-tNGS: Multiplex PCR-based targeted next-generation sequencing
MTBC: Mycobacterium tuberculosis complex
DTT: Dithiothreitol
NTC: Non-template control
RPhK: Reads per hundred thousand sequencing reads
ROC: Receiver operating characteristic
AUC: Area under the curve
PPV: Positive predictive value
NPV: Negative predictive value
CT: Computed tomography
HIV: Human immunodeficiency virus
MDR-TB: Multidrug-resistant tuberculosis
INH: Isoniazid
EMB: Ethambutol
DST: Drug susceptibility testing
CI: Confidence interval
SD: Standard deviation
κ: Kappa statistic
AIDS: Acquired immune deficiency syndrome
WBC: White blood cell
IL-6: Interleukin-6
ESR: Erythrocyte sedimentation rate
Hs-CRP: Hypersensitive C-reactive protein
LDH: Lactate dehydrogenase
PCT: Procalcitonin
NR: Normal range
PLB: Percutaneous lung biopsy
TBCB: Transbronchial cryobiopsy
TBB: Transbronchoscopic biopsy
TBNA: Transbronchial needle aspiration
TAT: Turnaround time.
